# A season for all things: Phenological imprints in Wikipedia usage and their relevance to conservation

**DOI:** 10.1371/journal.pbio.3000146

**Published:** 2019-03-05

**Authors:** John C. Mittermeier, Uri Roll, Thomas J. Matthews, Richard Grenyer

**Affiliations:** 1 School of Geography and the Environment, University of Oxford, Oxford, United Kingdom; 2 Mitrani Department of Desert Ecology, The Jacob Blaustein Institutes for Desert Research, Ben-Gurion University of the Negev, Midreshet Ben-Gurion, Israel; 3 School of Geography, Earth and Environmental Sciences, and Birmingham Institute of Forest Research, University of Birmingham, Edgbaston Birmingham, United Kingdom; 4 CE3C –Centre for Ecology, Evolution and Environmental Changes/Azorean Biodiversity Group and Univ. dos Açores–Depto de Ciências e Engenharia do Ambiente, Angra do Heroísmo, Açores, Portugal; Estacion Biologica de Doñana CSIC, SPAIN

## Abstract

Phenology plays an important role in many human–nature interactions, but these seasonal patterns are often overlooked in conservation. Here, we provide the first broad exploration of seasonal patterns of interest in nature across many species and cultures. Using data from Wikipedia, a large online encyclopedia, we analyzed 2.33 billion pageviews to articles for 31,751 species across 245 languages. We show that seasonality plays an important role in how and when people interact with plants and animals online. In total, over 25% of species in our data set exhibited a seasonal pattern in at least one of their language-edition pages, and seasonality is significantly more prevalent in pages for plants and animals than it is in a random selection of Wikipedia articles. Pageview seasonality varies across taxonomic clades in ways that reflect observable patterns in phenology, with groups such as insects and flowering plants having higher seasonality than mammals. Differences between Wikipedia language editions are significant; pages in languages spoken at higher latitudes exhibit greater seasonality overall, and species seldom show the same pattern across multiple language editions. These results have relevance to conservation policy formulation and to improving our understanding of what drives human interest in biodiversity.

## Abstract 2

Analysis of more than two billion page views over nearly three years for Wikipedia articles for 31,751 species across 245 languages reveals that more than a quarter of species show a seasonal pattern, and several online variations mirror real-world phenology.

## Author summary

Digital information archives offer novel opportunities to study human attitudes towards nature and to better understand how people interact with other species of animals and plants. The insights gained from such studies may be able to inform conservation efforts. Our study uses time-series of views to pages in the online encyclopedia Wikipedia to look at how human interest in other species varies seasonally across a wide range of different languages. In total, we extracted pageviews for 31,751 species of plants and animals across 245 Wikipedia language editions. Spanning nearly three years, our data set comprises 2.33 billion pageviews across 126,697 pages. We tested each time-series in our data set to see how well it fit a seasonal pattern and in doing so found several interesting patterns. First, seasonality is a significant factor in when people view information for many plants and animals online; over 20% of all of our species pages met our criteria for seasonality. Second, the prevalence of seasonality varies across different biological classes and also across languages. These variations appear to reflect differences in the life history of species and in the geographic distribution of languages and can correspond to phenological patterns in nature. Our results are relevant to conservationists seeking to understand how interest in various plants and animals may fluctuate over time.

## Introduction

Many aspects of human society respond to seasonal patterns. Seasonality can influence the foods people consume [1], the prevalence of diseases [2], and people’s mental health [3], among much else. Human interactions with biodiversity can also be seasonal; people may await the blooming of a flowering plant or expect the return of migrating birds at a particular season, and these phenological patterns can be an essential component of the “cultural value” of a species [4]. Despite this, seasonal patterns in human interest in biodiversity have received little attention in the conservation literature. Here, we demonstrate that identifying which species people interact with on a seasonal cycle and assessing whether those patterns are consistent across human geography and cultural groups is relevant to understanding what drives interest in biodiversity and can help promote improved conservation actions.

Public attitudes towards species can have a profound impact on conservation, with popular preferences for particular plants and animals potentially affecting the allocation of conservation resources by both governments and nongovernmental organizations (NGOs) [5,6] and determining the success and effectiveness of conservation initiatives [7,8]. Accordingly, it is important to understand what attributes of a species contribute to increased human interest [6,9], and how an organism’s popularity may vary as a result of factors such as a person’s nationality [10,11], socioeconomic status and age [12], education levels, and gender [13,14]. Seasonal changes in human interest in plants and animals can have an important role in conservation in at least three ways: (a) by identifying species for which phenology forms a component of their “value,” (b) by helping to reveal differences or similarities in how species are valued across cultural groups, and (c) by providing temporal awareness to help maximize the effectiveness of conservation marketing campaigns.

We analyze seasonal patterns in nearly three years of Wikipedia pageviews across 245 Wikipedia language editions for a large and taxonomically diverse group of species in order to explore (a) the prevalence of seasonal patterns in species pageviews, (b) differences in seasonal patterns across languages, (c) the effect of seasonality on the relative popularity of a species, and (d) the relationship between seasonal patterns in pageviews and phenology. As language is linked to human cultural identity and also has a geographic component, analyzing differences across languages provides insight into how seasonal patterns may vary culturally and geographically. Meanwhile, assessing the relationship between pageview seasonality and phenology helps in understanding the extent to which digital patterns parallel biological ones.

Large online data sets provide novel opportunities to examine interest in biodiversity at scales and resolutions that were previously inconceivable [15], and Wikipedia, the open-access encyclopedia, offers a unique resource that is well suited to making temporal comparisons in interest across large numbers of plants and animals. Wikipedia is currently the fifth most-visited website on the internet and, as of 2018, receives 14 to 16 billion pageviews per month across over 300 language editions [16,17]. Furthermore, Wikipedia’s structure, in which each page corresponds to a specific entity, facilitates the comparison of topics by avoiding semantic challenges such as homonyms [15,18]. Previous research has demonstrated that studying temporal trends in Wikipedia pageviews can provide insight into real-world phenomena [3,19,20]. Within conservation, Wikipedia pageviews have been used to compare human interest in reptile species [21]. As with any online data source, it is important to note that Wikipedia pageviews are not representative of all conservation stakeholders (people with limited access to the internet or those who live in countries where Wikipedia is blocked or unpopular are not represented), but pageview data do reflect the interests of a large and growing demographic that is of significant relevance to conservation.

## Results

### Prevalence of seasonality in online views

**Fig 1:**
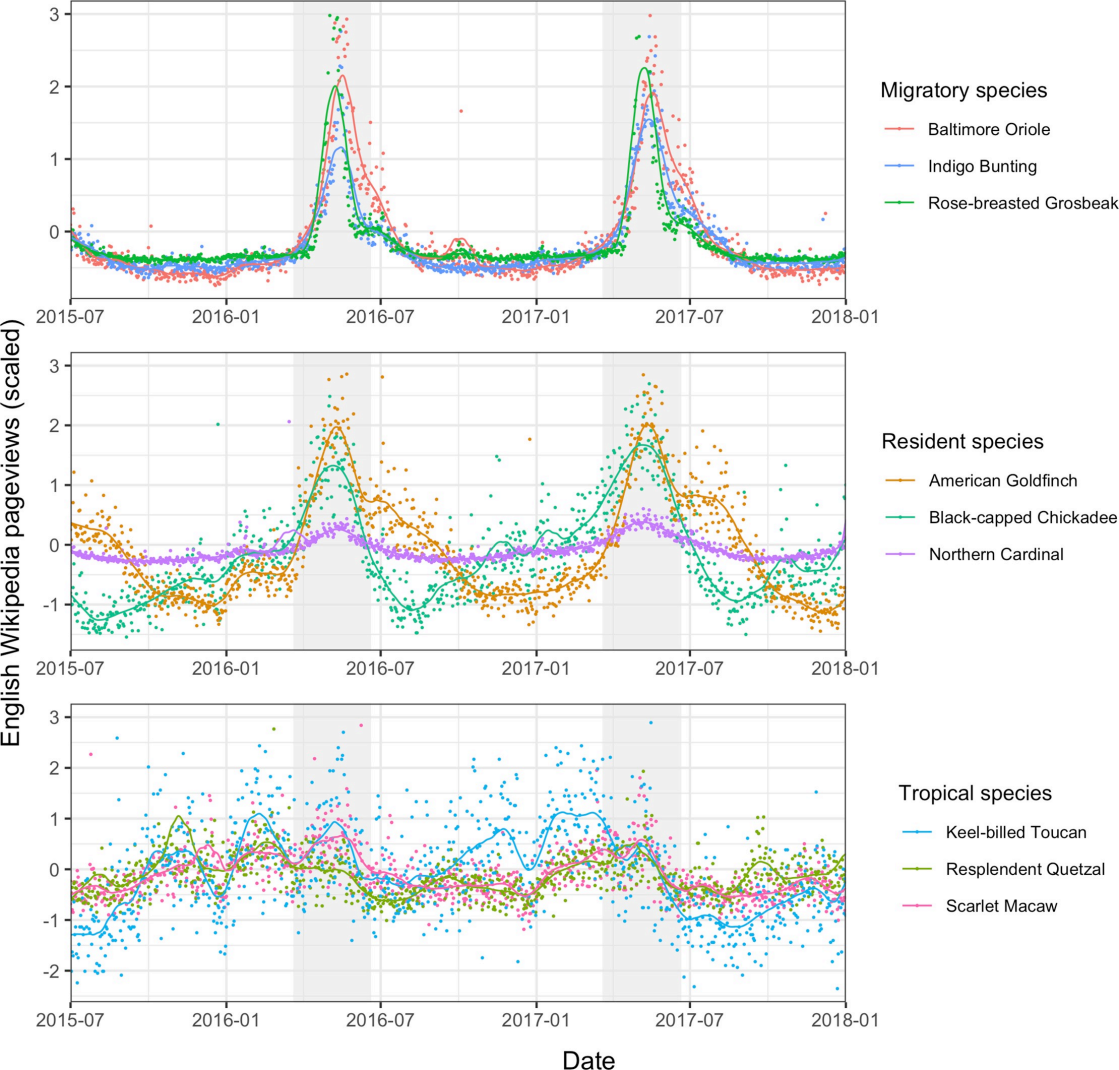
Daily pageviews in English-language Wikipedia for nine bird species. Pageviews for three migratory birds (top panel) show a strong seasonal peak coinciding with the bird’s arrival on breeding grounds in the United States. Pageviews for three North American resident birds show more variable patterns (second panel), and pageviews for three tropical species (bottom panel) that do not occur in the US show fluctuations over the course of the year but no consistent seasonality. Investigating the drivers of these patterns for individual species could be a rich area for further study, in particular as to whether changes in pageviews can be related to trends in population abundance and location or to particular human activities. Background gray shading indicates the dates of spring in the Northern Hemisphere. Pageviews for each species are scaled by subtracting the mean and dividing by the standard deviation (for data used in plots, see S1 Data).

**Fig 2:**
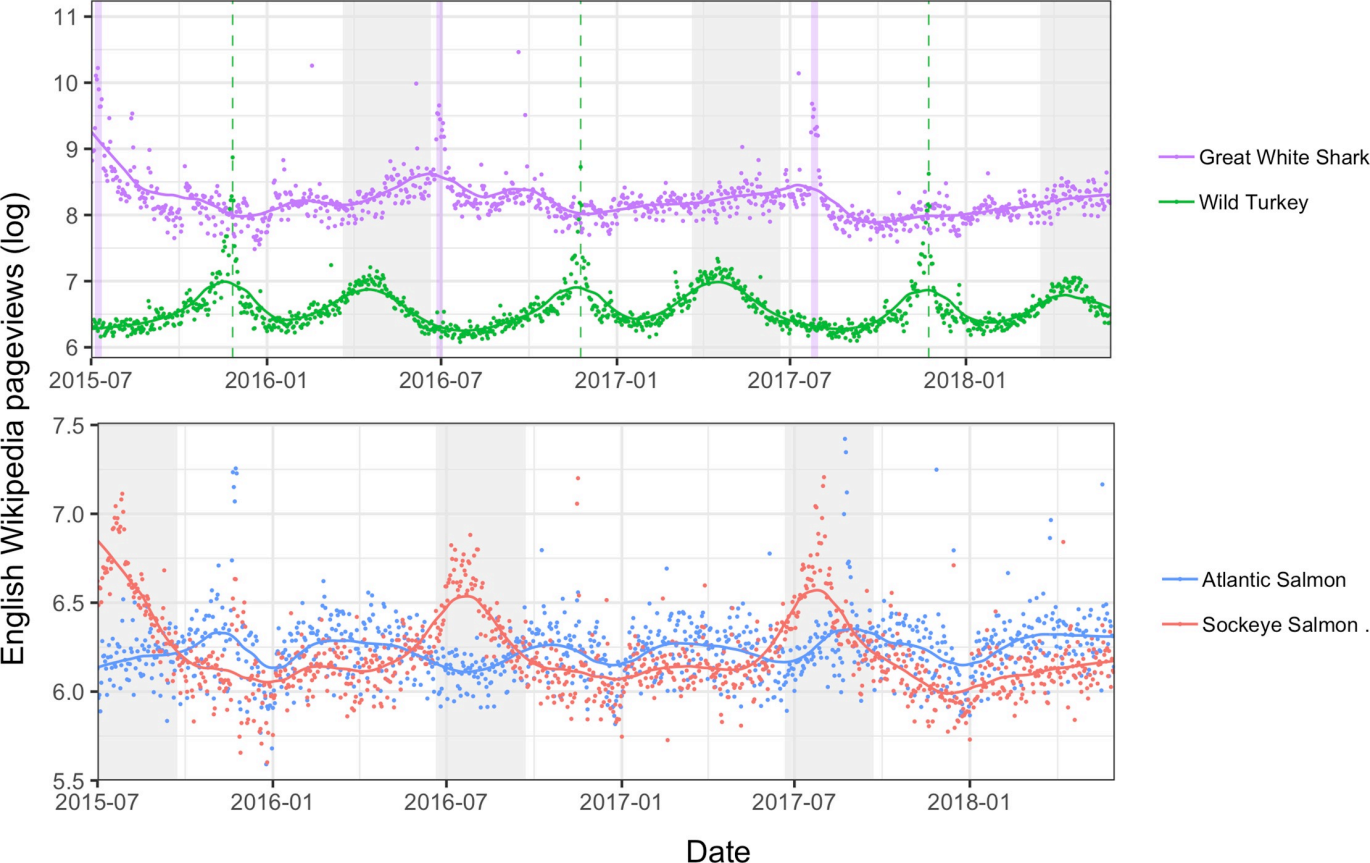
Effects of culture and phenology on the seasonality of interest in species. (Top panel) Patterns in Wikipedia pageviews respond to cultural influences as well as biological ones. English-language pageviews (logged) for great white shark *Carcharodon carcharias* (purple) are relatively stable throughout the year but show a brief spike during the days when “Shark Week” was aired on television by the Discovery Channel (days highlighted in purple). Pageviews for wild turkey *Meleagris gallopavo* (green) show a seasonal peak in the spring and a sharp peak during the Thanksgiving holiday in the US (date marked with a dashed line). The spring peak roughly coincides with the spring hunting season for wild turkey in many US states. (Bottom panel) The popularity of species relative to one another, as measured in Wikipedia pageviews, can vary as a result of seasonal fluctuations. Sockeye salmon *Oncorhynchus nerka* (red) and Atlantic salmon *Salmo salar* (blue) alternate in relative popularity depending on the time of year. Sockeye, which has a pronounced seasonal pattern, are more popular than Atlantic salmon in the Northern Hemisphere summer (dates shaded gray; for data used in plots, see S2 Data).

We identified seasonal patterns in Wikipedia pageviews in species pages (126,697 pages for 31,751 species) and a large sample of randomly selected nonspecies pages (121,638 pages for 9,158 entities). We assessed seasonality by comparing the fit (using adjusted R^2^ values) of detrended pageview data to sinusoidal models with either one or two annual peaks. A large proportion (20.2%) of pages in our species data set exhibit seasonal variation according to our criteria. This is significantly higher than among the randomly selected nonspecies pages, of which only 6.51% met our seasonality criteria (χ^2^_1_ = 10,083, *p* < 0.001). Adjusted R^2^ values for both single annual peak and double annual peak seasonal models were also significantly higher for species pages than for random pages. For the single annual peak model, for example, species-page mean adjusted R^2^ = 0.275 (standard error = 7.45 × 10^−4^), and random-page mean adjusted R^2^ = 0.131 (standard error = 5.56 × 10^−4^; Student *t*_230,730_ = −155, *p* < 0.001). In both species pages and random pages, the majority of seasonal pages fit a single annual peak (89.6% species, 82.5% random), with the remainder having two annual peaks. Aggregated at the species level, 25.2% of the species in our data set (8,015 of 31,751) show seasonality in at least one language edition. In many cases, seasonal patterns are striking and clearly correspond with phenological patterns (e.g., bird migration or breeding; Fig 1); however, seasonality also arises as a result of repeated cultural events, such as annual holidays (Fig 2).

**Fig 3:**
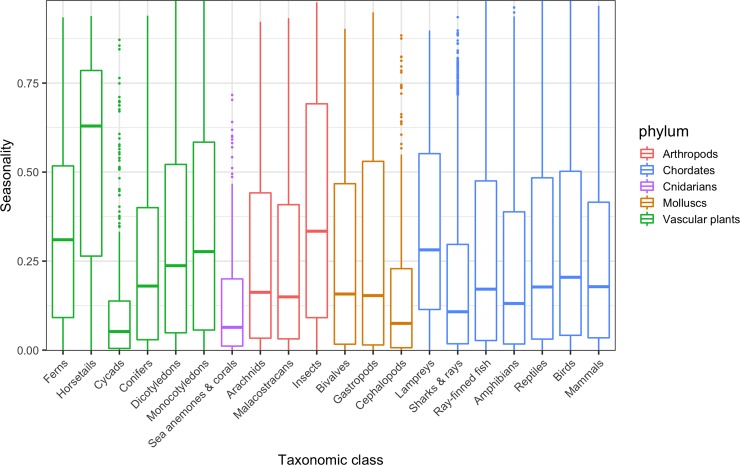
Prevalence of seasonality in Wikipedia pageviews varies across clades. Horizontal thick lines show median seasonality for species in 20 taxonomic classes in our data set that include over 100 species (*n* = 126,058 pages for 31,495 species). Bars indicate interquartile range, whiskers extend to 1.5× the interquartile range beyond the nearest quartile, and individual points are outliers beyond this. Seasonality is measured by the amount of variation in pageviews explained by a sinusoidal model with a single annual peak (via adjusted R^2^; for data used in plots, see S3 Data).

For taxonomic classes with at least 100 species in our data set (*n* = 20), the proportion of seasonal pages ranged from approximately 5% (Cycadopsida [cycads], Anthozoa [sea anemones and corals], Cephalopoda [squid and octopus]) to over 30% (Liliopsida [monocotyledons], Insecta [insects], Equisetopsida [horsetails]; Fig 3). For classes with greater than 1,000 species (*n* = 10), the most seasonal classes were insects (34.9% of pages) and monocotyledons (30.7% of pages), whereas the least seasonal were mammals (14.8%) and Elasmobranchii (sharks and rays, 9.31%).

### Variation across languages

Our data set of species pages includes pages from 245 Wikipedia language editions, and over half of the species in the data set (53.8%) appear in more than one language edition (mean pages per species = 3.99). Species tend not to show seasonality in all of the language editions in which they appear, even if they show strong seasonality in some, indicating that biogeographic and cultural complexity likely lies behind many interactions. For species that show seasonality in at least one language edition (*n =* 8,015), the mean percentage of seasonal pages per species is 40.7% (standard deviation = 21.9%), indicating that seasonal patterns are usually not consistent across languages. In only 126 cases (1.57%) is the same seasonal pattern (one or two peak) present across all language editions that a species occurs in, and in only 5 instances does this occur for a species that exists in more than five language editions (a migratory bird, two insects, and two flowering plants, all of them native to Europe).

**Fig 4:**
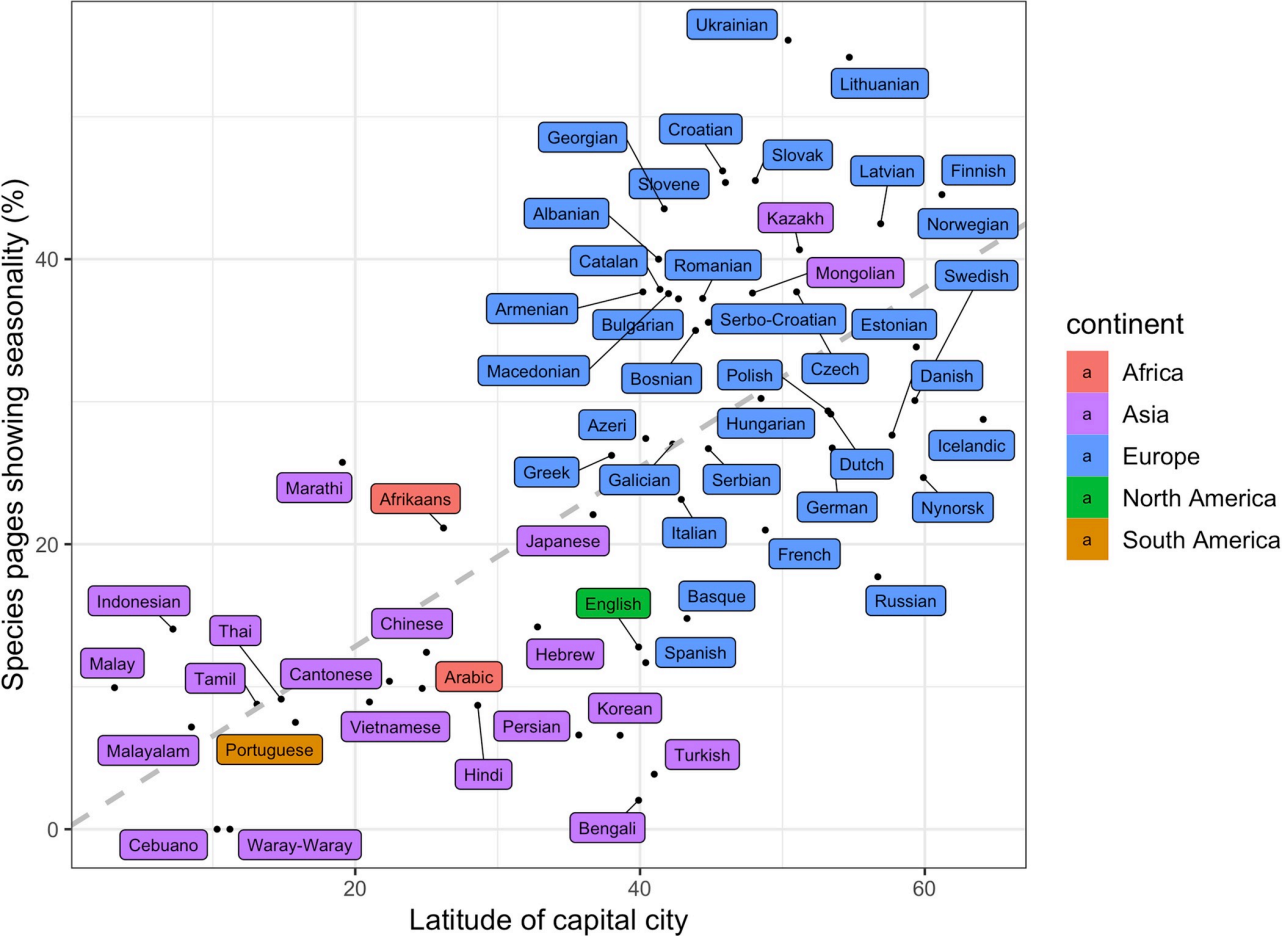
Seasonality and latitude of Wikipedia language editions. The percentage of seasonal pages for the 60 Wikipedia language editions in our data set that have over 100 species pages plotted against the absolute value of the latitude of the capital city of the country or province that accounts for the highest proportion of Wikipedia pageviews in that language (for data used in plots, see S4 Data).

For languages with at least 100 species pages (*n* = 60), the number of seasonal pages is uncorrelated with the total number of pages (*p* = 0.215, Pearson’s r = 9.4 × 10^−3^), indicating that smaller language editions are just as likely to have seasonality in their pageviews as larger ones. However, seasonality does show a significant positive relationship with capital city latitude (Fig 4; S1 Text), both when measured as the percentage of seasonal pages in a language (percent seasonal pages = 0.63 × |latitude| + 0.24, *p* < 0.001, adjusted R^2^ = 0.44) and when calculated as the mean seasonality of all pages fitting a single seasonal peak in a language (mean seasonality = 4.5 × 10^−3^ × |latitude| + 0.13, *p* < 0.001, adjusted R^2^ = 0.48).

### Impact of seasonality on relative popularity

Monthly rankings in the total pageviews for a species varied over the temporal period of our dataset (mean standard deviation = 1,850 ranks), with the average species shifting in its popularity (as measured by summed pageviews) relative to other species by 5.8%. As is to be expected statistically, these fluctuations were more pronounced for species with fewer views overall (mean standard deviation lower pageview quartile = 2,660 ranks; upper quartile = 520 ranks), indicating that relative popularity is more stable for the species that receive more views. Nevertheless, temporal fluctuations shifted the relative popularity of species over the course of a year even for some of the most viewed species overall.

### Correspondence between pageview seasonality and eBird frequency

We used bird frequency records from four countries (Italy, Germany, Sweden, and the US), extracted from the citizen science database eBird.org, to correlate annual variations in the frequency with which a bird is recorded in a country with seasonal patterns in Wikipedia pageviews in that country’s national language. For the 862 pages that we tested across our four target languages, 54.1% (466 pages) showed a significant relationship between monthly Wikipedia pageviews and monthly eBird frequency (*p* < 0.05), and 87.6% of these (408 pages; 47.3% of all pages) showed a significant positive relationship between the two (mean scaled coefficient = 0.640, mean adjusted R^2^ = 0.42; S1 Fig). The proportion of bird pages in our data set with a significant positive relationship between monthly eBird frequency and monthly pageviews was relatively consistent across the four countries—49.1% of species in the US, 48.9% in Germany, 45.4% in Sweden, and 39.8% in Italy. For species that occurred in more than one of the four languages and/or countries evaluated (*n =* 146), only 34.2% (*n* = 50) showed a significant positive relationship between eBird frequency and pageviews across multiple languages. Species that were more seasonal in their pageviews (i.e., had a better fit to the seasonal model) were more likely to have a positive relationship between eBird frequency and pageviews (coefficient = 2.09, standard error = 0.497, *p* < 0.001). In contrast, the total number of views that a page received was not a good predictor of whether there was a positive relationship between pageviews and eBird frequency (coefficient = 1.13 × 10^−6^, standard error = 4.65 × 10^−7^, *p* = 0.015).

## Discussion

Using a large, online encyclopedia, we provide the first broad exploration of seasonal patterns of interest in nature across many species and cultures. We show that seasonal patterns play a widespread and significant role in online interest in plants and animals, with a quarter of the 31,751 species in our data set exhibiting seasonal patterns in at least one language. Wikipedia, and internet usage in general, may respond to seasonal fluctuations; however, the fact that seasonality is significantly more prevalent in species pages than among a random selection of Wikipedia articles suggests that human interactions with nature are particularly likely to be seasonal. The drivers of these seasonal patterns are complex and variable (e.g., Figs 1 and 2). As evidenced by the bird species in our eBird sample, however, seasonality in online interest can often have a positive relationship with phenological patterns, such as variations in the local presence and abundance of a species. The variation in pageview seasonality across taxonomic clades also suggests links to phenology. Insects and flowering plants—for example, clades whose species frequently have conspicuous phenological variations—display higher seasonality than two clades of plants (cycads and conifers) and several groups of marine organisms (sharks and rays, sea anemones and corals, squid and octopus), in which phenological variations or reproductive events are likely to be less visible to the nonspecialist (Fig 3).

These broad patterns in seasonality can have direct relevance for conservation practice. Sharp and predictable temporal spikes in interest create a clear opportunity for NGOs seeking to maximize the impact of their fundraising campaigns. Exploring the existence of temporal trends in interest in a specific target species and investigating what drives those trends should be an initial first step when setting up fundraising campaigns. For this purpose, the methodology we present here could aid in effectively identifying and targeting flagship species [22]. Furthermore, the seasonal variations in relative popularity across species is a relevant methodological consideration for researchers comparing interest across sets of species, particularly when using online behavior as a proxy for interest or popularity. As we demonstrate, online views for some species can fluctuate significantly on an annual cycle, and these variations could be a confounding variable in investigating other drivers of interest. For example, the online popularity of two salmon species depends, in part, on when the data are collected (Fig 2). These fluctuations are particularly pronounced for less popular species but also impact highly viewed ones.

We also demonstrate the high importance of geographic and cultural differences (in our case, exemplified in language editions) in human interactions with other plants and animals. In our data, this is reflected in the fact that species seldom show the same seasonal pattern across Wikipedia language editions. It is important to note, however, that the potential drivers of these differences are complex. Although language-level differences in pageviews can result from biological or cultural factors, they can also arise from differences in the structure of Wikipedia. Language editions vary vastly in size; a minority have millions of articles and users, whereas most have considerably fewer. The fact that we were able to detect predictable variation in seasonality between languages despite these differences (Fig 4) underscores the significance of seasonal patterns. Being aware of these seasonal patterns can benefit conservation campaigns, efforts to raise awareness, and educational endeavors. With the addition of other more precisely geolocated digital tools, it would be possible to identify (and manage) areas where people go to observe particular phenological phenomena (e.g., [23]).

Overall, we highlight the utility of culturomic tools in broad explorations of the seasonal aspect of human–nature interactions and demonstrate how phenology, geography, and culture can play intricate roles when exploring the seasonality of interest in nature. There is fertile ground for further study of some of the patterns that appear in this initial exploration. As always, drawing broad conclusions when using these tools should be done with caution, particularly if generalizing beyond the specific user base of the online resource [15]. Nevertheless, we feel that these methods hold much promise in elucidating the seasonal component of human–nature interactions, which has, to date, been understudied and underappreciated.

## Materials and methods

### Data

Our data set comprises 2.33 billion pageviews for 126,697 pages across 245 Wikipedia languages with each page having a corresponding time-series of daily pageviews over 1,067 days. The data set contained pages for 31,751 species representing 52 taxonomic classes and 1,611 families. Our list of species and subspecies, representing a wide range of taxa, was compiled from Wikidata, a document-oriented database that provides data to projects run by the Wikimedia foundation. Using the Wikidata Query Service (https://query.wikidata.org), we extracted items that had both a Global Biodiversity Information Facility ID (identifier: P846) and an International Union for Conservation of Nature (IUCN) conservation status (statement: P141) on 05 June 2018. This two-part validation helped to control against items tagged erroneously. Additional taxonomic data (scientific name, higher taxa information) for each of these was compiled using the package “rgbif”[24] in R [25], and corresponding Wikipedia pages were extracted using “rvest” [26]. Pageviews for the period between 01 July 2015 and 02 June 2018 were summarized with “pageviews” [27]. These dates were selected to maximize the time frame of our data set; 01 July 2015 is the date that Wikipedia began its current system for archiving pageview data, and thus the earliest date for which we could extract pageviews with these methods, and 02 June 2018 was the date of our data extraction. To assess seasonality, we restricted our analyses to pages that received, on average, at least one view per day (using Tukey’s biweight mean; package “dplR”[28]). We used these same methods to extract a comparative data set of 2.45 billion pageviews for 121,638 pages for 9,158 randomly selected nonspecies Wikidata entities across 290 Wikipedia languages. Random pages were selected by generating 10,000 random integers between 0 and 1 million (sample.int function in R) and then converting these into Wikidata entity IDs. The resulting Wikidata entities were cross-referenced against our list of species, and species pages were removed from the random sample.

### Determination of seasonality

For each series of pageviews (corresponding to one Wikipedia page) in our data sets, we fitted a locally estimated scatterplot smoothing (LOESS) model to the logged daily views using the R package ‘fANCOVA” [29]. Smoothing parameters for the LOESS were chosen using the Akaike Information Criterion corrected for small sample size (AICc [30]). We then used this model’s fitted values to test for seasonality by (a) identifying annual consistency in the time-series by testing the fit between the data from the first year (days 1–365) and the second year (days 366–730) and (b) fitting sinusoidal models with one or two annual peaks to the detrended (residuals of a linear model) daily views of each time-series. The first two years of the data were used for testing the model, because these were complete cycles of 365 days (the third year was approximately one month short of a full year due to the timing of our study). We used a linear model rather than a simple null model in order to account for overall growth in the size and use of Wikipedia over the sample period. We used a log-likelihood test [31] to select between the two seasonal models and set thresholds based on adjusted R^2^ to classify whether a time-series was consistent (year 1 pageviews predicted year 2 pageviews with adjusted R^2^ > 0.5) and whether it fit the sinusoidal model (adjusted R^2^ > 0.5 for time-series fitting a single annual peak, and > 0.3 for a double annual peak). The selected thresholds for adjusted R^2^ were derived by manually reviewing the data and comparing results to well-established seasonal patterns, such as the annual variation in day length. Sensitivity testing with lower (adjusted R^2^ > 0.3) and higher thresholds (adjusted R^2^ > 0.7) varied the results in a predictable manner but did not significantly alter the relative proportion of seasonal patterns relative to each other within species pages or between species pages and random pages (S1 Text). Initial tests that used sinusoidal models with three and four annual peaks were never selected by the log-likelihood test, and these models were excluded from the final analysis. A time-series met our criteria for being seasonal if it passed both the thresholds for annual consistency and the best-fitting sinusoidal model (with either one or two peaks).

### Linguistic variation in Wikipedia pageviews

Wikipedia language editions vary greatly in their total pages, and as expected, our data included a large range in the number of species pages per language (range 1–28,393; mean 517). For languages that contained more than 100 species pages (*n* = 60), we correlated pageview seasonality with language edition size (measured by the total number of species pages in the language) and modeled the relationship between pageview seasonality and the absolute value of the latitude of the capital city of the country that accounts for the highest percentage of Wikipedia pageviews in that language (e.g., Portuguese → Brazil → Brasilia → 15.8° S; Wikipedia views by country from [32]; see S1 Text for details). We explored temporal trends in the rank popularity of species by summarizing the monthly views across all of the pages for a species and calculating its monthly ranking against other species in the data set.

### Concordance between Wikipedia seasonality and eBird frequency data

We extracted bird frequency records for Italy, Germany, Sweden, and the US from eBird.org [33] using “rebird” [34] (see S1 Text for details). For bird pages in the relevant language for each of the four countries (Italian, German, Swedish, and English, respectively), we extracted pages that (a) met our criteria for pageview seasonality and (b) covered a species that occurred in the country in question and therefore had an eBird frequency distribution (species per country: US, *n* = 472; Italy, *n =* 108; Germany, *n* = 141; Sweden, *n =* 141). Accordingly, each of the 862 pages in our data set corresponds to a specific species and country, and species that occur in more than one country are represented by separate pages for each country. For each page, we used a linear model to assess the relationship between monthly eBird frequency and monthly pageview totals. Significance values were adjusted using the false discovery rate for multiple comparisons [35].

## Supporting information

S1 Text Additional information on methods to establish thresholds for seasonality, assign latitude to Wikipedia languages, and compare eBird frequency and Wikipedia pageview data. (DOCX)

S1 Fig Histogram of the distribution of significant correlation coefficients (FDR-adjusted *p* < 0.05) between monthly eBird frequency and monthly pageviews to seasonal Wikipedia pages for bird species that occur in Germany, Sweden, Italy, and the US. The distribution of values indicates that monthly increases in pageviews often correspond temporally with increased observations of a bird species in a given country. FDR, false discovery rate.
(TIF)

S1 Data Data used in Fig 1. (CSV)

S2 Data Data used in Fig 2. (CSV)

S3 Data Data used in Fig 3. (CSV)

S4 Data Data used in Fig 4. (CSV)

## Abbreviations

AICc: Akaike Information Criterion corrected
IUCN: International Union for Conservation of Nature
NGO: nongovernmental organization
