# Supplementary material for: A season for all things: Phenological imprints in Wikipedia usage and their relevance to conservation
Source: PLoS Biol. 2019 Mar 5;17(3):e3000146. doi: 10.1371/journal.pbio.3000146 (PMC6400330; doi:10.1371/journal.pbio.3000146)
Supplement: S1 Text — (DOCX) [file pbio.3000146.s001.docx]

**S1 Text**

**Sensitivity testing of seasonality threshold**

We qualified a time-series as being seasonal based on: a) the consistency between year 1 and year 2 of our data, and b) the fit between a sinusoidal model containing one or two annual peaks and the detrended (residuals of a linear model) daily views. We used thresholds in adjusted R^2^ to classify whether a time-series was consistent (year 1 pageviews predicted year 2 pageviews with adjusted R^2^ > .5) and whether it fit the sinusoidal model (adjusted R^2^ > .5 for time-series fitting a single annual peak, and > .3 for a double annual peak). Since there are no quantitative definitions of which we are aware for classifying time-series in this way, we derived our thresholds by plotting and manually comparing different time-series. We sensitivity tested our chosen thresholds by comparing the proportion of seasonal pages in our species and random datasets at lower (adjusted R^2^ > .3 for time-series fitting a single annual peak, and > .1 for a double annual peak) and higher thresholds (adjusted R^2^ > .7 for single annual peak, and > .5 for a double annual peak). These adjustments shifted the total number of pages meeting the criteria for seasonality in a predictable fashion given the distribution of adjusted R^2^ values in the data. For example, at the threshold we selected, 20.2% of pages in the species dataset qualified as seasonal; at the lower sensitivity this increased to 25.62%, and at the high sensitivity it dropped to 9.71%. Changes in the values for both species- and random-pages at different thresholds are listed below.

|  | Species pages | | | Random pages | | |
| --- | --- | --- | --- | --- | --- | --- |
|  | low | middle | high | low | middle | high |
| Pages with one peak (%) | 23.16 | 18.09 | 9.1 | 8.88 | 5.40 | 1.72 |
| Pages with two peaks (%) | 2.46 | 2.10 | 0.6 | 1.41 | 1.11 | 0.25 |
| Total seasonal pages (%) | 25.62 | 20.20 | 9.71 | 10.28 | 6.51 | 1.97 |

**Assignment of latitude to Wikipedia languages**

We used the latitude of the capital city of the country that accounted for highest proportion of Wikipedia page views in a given language as a proxy for the latitudinal distribution of Wikipedia users of that language. For example: Portuguese -> Brazil -> Brasilia -> 15.8 ° S. Clearly, mapping the linguistic distribution of languages, particularly those with large numbers of speakers, is complex. Furthermore, the distribution of Wikipedia users is unlikely to be evenly distributed amongst the population of a language’s speakers. In this context, our approach obviously provides only a rough approximation of linguistic distribution. In addition to other complicating factors, it varies in its precision across languages. Spanish Wikipedia, for example, receives the most visits from Spain, and so the latitude of Madrid was counted in our distributions. Spain’s margin of views over other countries, however, is small, and a large proportion of Spanish Wikipedia views come from other countries (Spain 22.1%, Mexico 19.8%, Argentina 12.3% of total views). In Japanese Wikipedia, by contrast, 96.1% of views come from Japan. Despite these drawbacks, we argue that this method nevertheless offers useful insight when making very broad-scale comparisons between the latitudinal distributions of languages.

**Pageview and eBird data for target countries**

We selected four target countries to compare Wikipedia pageviews to eBird frequency data. Three European countries were selected on the basis of having large Wikipedia editions with a high proportion of the pageviews originating in a single language (Italian 86.6% from Italy, German 77.2% from Germany, Swedish 89.0% from Sweden [1]). The United States, which accounts for only 41.5% of English Wikipedia views [1], was also included because it has an exceptionally large dataset both in total pageviews and total bird records. The frequency of a species in eBird is derived from the proportion of checklists mentioning that species, and provides a conservative metric of the likelihood of encountering a species at a given time and location [2]. In order to obtain a robust sample of frequency on annual scale we summed monthly frequency across all years from 1900-2018. Total eBird records for each of the target countries were as follows: Italy *n* = 9.5k checklists; Germany 24.9k; Sweden 22.4k; United States 19.2M.

References

1. Zachte E. Wikimedia Traffic Analysis Report: page views per wikipedia language [Internet]. 2018 [cited 2018 Jul 23]. Available from: https://stats.wikimedia.org/wikimedia/squids/SquidReportPageViewsPerLanguageBreakdown.htm

2. Sullivan BL, Wood CL, Iliff MJ, Bonney RE, Fink D, Kelling S. eBird: A citizen-based bird observation network in the biological sciences. Biol Conserv [Internet]. Elsevier Ltd; 2009;142(10):2282–92. Available from: http://dx.doi.org/10.1016/j.biocon.2009.05.006
