# Supplementary figures and images for: A season for all things: Phenological imprints in Wikipedia usage and their relevance to conservation
Source: PLoS Biol. 2019 Mar 5;17(3):e3000146. doi: 10.1371/journal.pbio.3000146 (PMC6400330; doi:10.1371/journal.pbio.3000146)

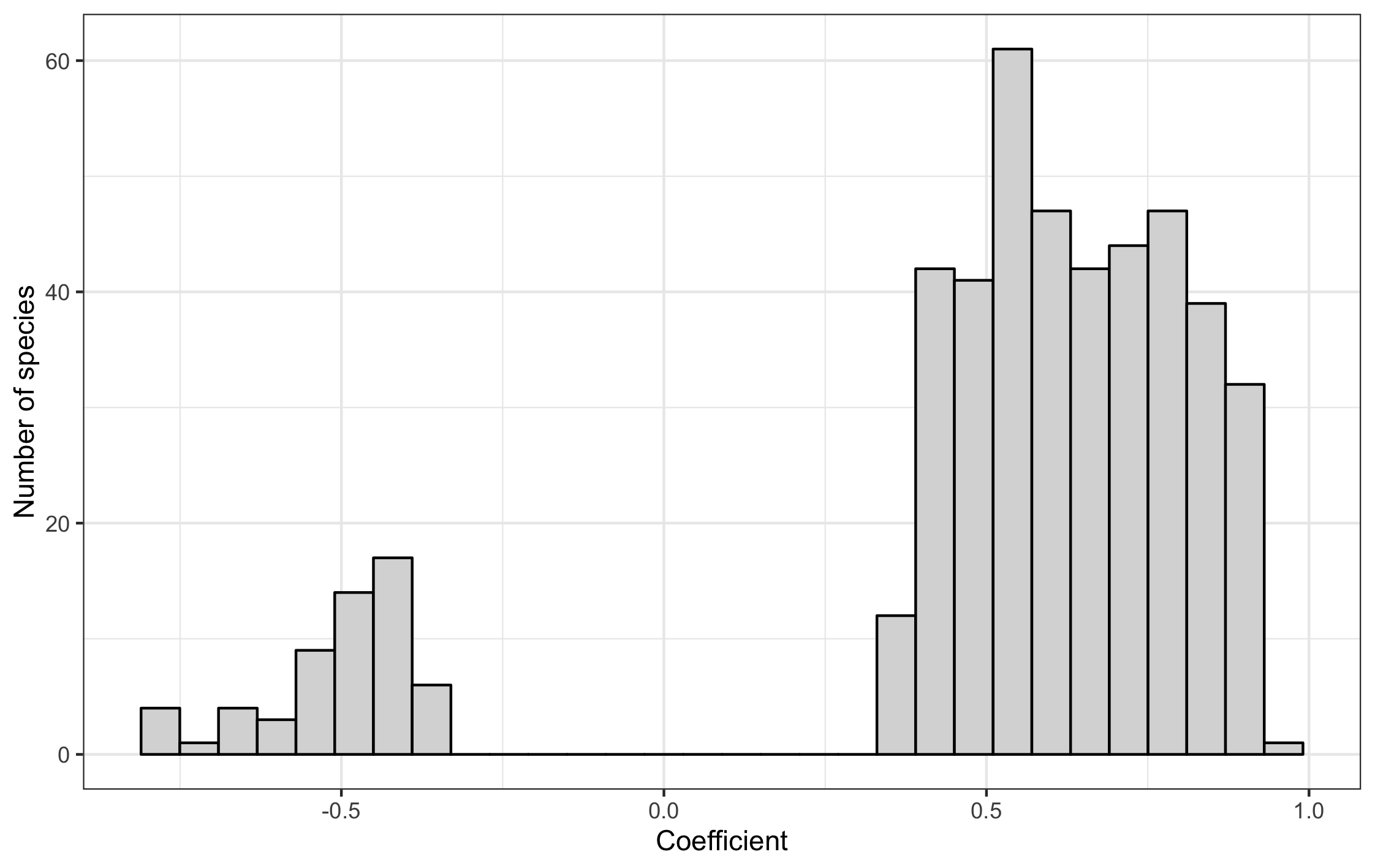

Supplement: S1 Fig — The distribution of values indicates that monthly increases in pageviews often correspond temporally with increased observations of a bird species in a given country. FDR, false discovery rate. (TIF) [file pbio.3000146.s002.tif]
